# Effects of lysophospholipid supplementation in diets with different oil sources on broiler performance under heat stress

**DOI:** 10.3389/fvets.2025.1561679

**Published:** 2025-04-24

**Authors:** Maziar Mohiti-Asli, Moin Ghanaatparast-Rashti, Seyed Davood Sharifi, Hasan Rouhanipour, Pooya Akbarian

**Affiliations:** ^1^Department of Animal Science, Faculty of Agricultural Sciences, University of Guilan, Rasht, Iran; ^2^Department of Animal and Poultry Science, Faculty of Agricultural Technology, University of Tehran, Tehran, Iran; ^3^Pathway Intermediates, Seoul, Republic of Korea

**Keywords:** broilers, growth performance, lysolecithin, nutrient digestibility, vegetable oil

## Abstract

**Introduction:**

Feed cost has gradually increased in poultry production worldwide. One approach to minimizing production cost is dietary manipulation of nutrient supplies through improved feed efficiency. The inclusion of lipids in broiler diets is essential to meet metabolisable energy requirement. This study aimed to investigate the effects of adding lysophospholipids (LP) to diets varying in sources and levels of vegetable oils on growth performance, ileal digestibility of nutrients, and serum lipids of heat-stressed broilers.

**Methods:**

A total of 320 Ross 308 broiler chicks were used in a completely randomized design with a 2 × 2 × 2 factorial arrangement of two oil sources (soybean oil; SO, and palm oil; PO), two levels of oil (3 and 6%), and two levels of LP (0 and 100 mg/kg), with four replicates and 10 birds in each replicate. From 28 to 42 days of age, broilers were exposed to 36°C for 5 h everyday.

**Results:**

Supplementation of LP to broilers diet increased BWG from 1 to 21 d of age and for the whole period (*p <* 0.05). Broilers fed diet containing 6% oil with LP had lower FI from d 22 to 42 d, and better FCR from 22 to 42 d of age and over the entire period (*p <* 0.05). Apparent ileal digestibility (AID) of EE, and AIDE was higher in diets containing 6% oil than diets with 3% oil (*p <* 0.05). The AID of EE, and AIDE were higher in broilers fed LP in the diets containing 6% oil (*p <* 0.05). The LP supplementation to diets reduced serum TG, total cholesterol, and LDL-C, whereas enhanced HDL-C concentrations at 42 d of age (*p <* 0.05).

**Conclusion:**

It was concluded that broilers fed diets containing SO performed better, and LP supplementation enhanced the performance of broilers fed diets containing higher levels of oils.

## Highlights

- Broiler chickens fed soybean oil had better weight gain, feed intake, and feed conversion ratio than those fed palm oil.
- Dietary emulsifiers enhance nutrient digestion, and growth performance, particularly in the early stages of rearing.
- Lysophospholipid emulsifiers enhance growth performance and nutrient digestibility in broilers chickens, especially under challenging conditions like heat stress.
- Lysophospholipid emulsifiers benefit broiler chickens’ growth, and nutrient digestion, under heat stress condition.

## 1 Introduction

Hot climate is a worldwide problem that causes poor performance and loss of economic benefits, particularly after the third or fourth week of age, Especially Heat stress (1). Heat stress represents a challenge to poultry production worldwide and has results in behavioral and physiological changes in poultry and has negative effects on health, productivity and product quality (2). The adverse effects of heat stress on poultry can be briefly described as reduced feed intake (3), which is the major reason of decreased weight gain, increased mortality, reduced fertility and hatchability, alteration of the electrolyte balance and blood pH, impaired secretion and endogenous enzymes activity, decreased serum concentrations of tri-iodothyronine (T3) and tetra-iodothyronine (T4), suppressed immune function and decreased intestine absorbing capacity. Various methods have been established to prevent and alleviate heat stress in birds (4).

Broilers must receive adequate energy and protein to fully explicit their genetic potential. There is also considerable evidence suggesting that increasing energy concentration by using a larger lipids/oils content enhances the development performance of birds exposed to heat stress (5, 6). Therefore, large quantities of animal lipids and vegetable oils have the highest caloric density and are added to broiler diets to increase energy content (7). Lipids are water-insoluble molecules that are digested by a synergistic action of pancreatic lipase and bile salts. Lipid digestion occurs in an aqueous environment of the intestinal lumen, making bile salts necessary for the efficient emulsification of dietary lipids. This lets pancreatic lipase hydrolyze the triglycerides present at the water–oil interface, leading to free fatty acids and mono-glycerides (8). Bile salts are also involved in the organization of mixed micelles, which are then attracted by the small intestine mucous membrane. Overall, lipid digestion and absorption are a complex action that requires an enough amount of bile salts, pancreatic lipase, and co-lipase. In the absence of even one of these essential substances, digestion, and absorption are disturbed.

Attention to several factors can be necessary for lipid digestion, including bird specifications like the age, genetics, secretion, and activity of digestive enzymes, microflora status, and diet fat consumed (9). Since broiler diets have a high lipid content, a potential strategy to improve lipid digestibility and poultry performance is to consider the use of exogenous emulsifiers for both emulsion and micelle formation processes.

A variety of emulsifier feed additives are commercially available for use in animal diets, which are classified into three types: lysolecithins, crude lecithins, and synthetic blends (glycerol polyethylene glycol ricinolate). It is important to consider the ability of the emulsifier to dissolve water or fat (10), which is evaluated as hydrophilic–lipophilic balance (HLB). Lysophospholipids (LP) are produced by the catalytic activity of phospholipase-A2 on soy-lecithin and it has higher hydrophilic properties than normal phospholipids (11). LP has a critical micelle concentration that is 20–200 times lower than bile acid and lecithin (12, 13). As a result, LP supplementation appears to improve digestion, dietary fat absorption, and productive performance in animals. Albeit, the positive effects of LP in broiler diets have been reported (14, 15), but the data found in the literature cannot be used to determine their efficacy due to differences in the formulation of basal diets.

To our knowledge, there are no signs of the need to match the level of lysophospholipid content in response to increasing the fat content of diets at different phases of feeding. The objective of this study was to investigate the interaction of LP supplementation and sources and levels of vegetable oils in diets on growth performance, ileal digestibility of nutrients, and serum lipid parameters of heat-stressed broilers.

## 2 Materials and methods

### 2.1 Animal ethics

The experiment was conducted in the Poultry Research Station at University of Guilan, and the whole of the experimental methods utilized in the present study were approved by the University’s Animal Ethics Committee.

### 2.2 Birds, experimental design, and diets

A population of 320 one-day-old Ross 308 broiler chicks with an average body weight of 43.8 ± 0.7 g were used uniformly by sex and weight into floor pens (1 m × 1 m). The broilers were randomly assigned to a 2 × 2 × 2 factorial arrangement of diets with two different sources of oil (soybean and palm), two levels of oil (3 and 6%), and two inclusion rates of LP (Lipidol; 0 and 100 mg/kg; Pathway Intermediates, Seoul, South Korea), with four replicates per treatment and 10 broilers per floor pen. Lysophospholipids (LP) were supplemented to broiler diets in the form of a commercial lysolecithin product containing 4.06% of a standardized mixture of lysophosphatidylcholine, lysophosphatidylethanolamine, lysophosphatidylinositol and lysophosphatidic acid. The temperature was set at 32°C for the first week of age and then decreased to 23°C until 21 days of age. At the ages of 28 to 42 days, the broilers in the heat stress (HS) group were reared in 34 ± 1°C for 8 h daily while the following temperature was set up during 2 h (between 8:00 a.m. and 10:00 a.m.) and gradually increased to 36°C. This high temperature was then maintained for 8 h (until 16:00 p.m.). After that, the temperature was gradually decreased to the basal level (18:00 p.m.). The relative humidity was kept between 60–70% during the experimental period. Cloacal temperature was measured just after exposure to HS by using a digital thermometer with an accuracy of 0.1°C, using one chick per replicate. The respiration rate just after exposure to HS was estimated by counting the breaths/min by watching movement of the abdomen for a minute. In the first 2 days light regimen was continuous with an intensity 20 lux and thereafter, a 23 L: 1D lighting schedule was used with 5 lux for the remaining period (3–42 days). All broilers were fed with Ross 308 recommendations (16). The ingredients and calculated nutrient composition of the basal diets are presented in Table 1. The feed in mash form and fresh water were provided *ad libitum* to the broilers throughout the entire duration of the experiment.

**Table 1:** Ingredient and nutrient composition of the basal diets (%, as fed basis).

Items	Starter (1–10 d)				Grower (11–24 d)				Finisher (24–42 d)			
	Soybean oil (%)		Palm oil (%)		Soybean oil (%)		Palm oil (%)		Soybean oil (%)		Palm oil (%)	
	3	6	3	6	3	6	3	6	3	6	3	6
Ingredient (%)												
Maize, 7/20% CP	50.91	39.15	53.23	43.95	56.44	44.66	56.96	45.82	61.38	48.85	62.80	51.56
Soybean meal, 44/54% CP	37.30	36.65	37.88	37.43	33.89	32.17	34.02	32.51	27.55	26.29	27.96	26.50
Wheat bran, 16/37% CP	4.30	13.75	1.40	8.16	0.66	11.16	0.00	9.66	4.33	15.00	2.51	12.20
Vegetable oil	3.00	6.00	3.00	6.00	3.00	6.00	3.00	6.00	3.00	6.00	3.00	6.00
Dicalcium phosphate	2.01	1.92	2.03	1.97	1.83	1.75	1.84	1.76	1.63	1.54	1.64	1.56
Calcium carbonate	0.95	0.99	0.94	0.96	0.82	0.86	0.82	0.85	0.77	0.94	0.76	0.80
Common salt	0.23	0.22	0.23	0.23	0.26	0.24	0.26	0.24	0.24	0.23	0.25	0.23
Sodium bicarbonate	0.15	0.16	0.15	0.15	0.11	0.13	0.11	0.13	0.13	0.14	0.12	0.14
Celite	0.00	0.00	0.00	0.00	2.00	2.00	2.00	2.00	0.00	0.00	0.00	0.00
L-Lysine HCl, 78%	0.24	0.23	0.23	0.23	0.17	0.19	0.17	0.19	0.19	0.20	0.18	0.20
DL-Methionine, 98%	0.31	0.32	0.31	0.32	0.26	0.27	0.26	0.27	0.23	0.24	0.23	0.24
L-Threonine, 99%	0.10	0.11	0.10	0.10	0.06	0.07	0.06	0.07	0.05	0.07	0.05	0.07
Vitamin Premix^1^	0.25	0.25	0.25	0.25	0.25	0.25	0.25	0.25	0.25	0.25	0.25	0.25
Mineral Premix^2^	0.25	0.25	0.25	0.25	0.25	0.25	0.25	0.25	0.25	0.25	0.25	0.25
Total	100	100	100	100	100	100	100	100	100	100	100	100
Calculated Analysis	Starter (1–10 d)				Grower (11–24 d)				Finisher (24–42 d)			
Metabolizable Energy, kcal/kg^−1^	2,900				2,950				3,050			
Crude Protein, %	22.02				20.11				18.33			
Digestible Lysine, %	1.22				1.08				0.97			
Digestible Methionine, %	0.61				0.53				0.49			
Digestible Methionine + Cystine, %	0.90				0.81				0.75			
Digestible Threonine, %	0.82				0.72				0.65			
Calcium, %	0.91				0.81				0.75			
Available Phosphorus, %	0.48				0.44				0.40			
Sodium, %	0.15				0.15				0.15			
Potassium, %	0.94				0.85				0.80			
Chloride, %	0.23				0.23				0.23			
Analyzed composition^3^												
Dry Matter, %	88.34	88.50	88.48	88.61	88.25	88.72	88.43	88.55	88.57	88.64	88.45	88.57
Gross energy, kcal kg^−1^	4,175	4,373	4,168	4,354	4,234	4,421	4,223	4,417	4,288	4,476	4,283	4,473
Crude protein (Nitrogen × 6.25), %	21.67	21.72	21.70	21.65	19.53	19.62	19.66	19.58	17.92	17.95	17.98	17.94
Ether extract, %	5.82	8.66	5.87	8.74	5.76	8.72	5.91	8.79	5.94	8.86	5.97	8.80

1 Vitamin premix provided the following per kilogram of diet: vitamin A (*trans*-retinyl acetate), 10,000 IU; vitamin D_3_ (cholecalciferol), 2000 IU; vitamin E (DL- *α*-tocopherol acetate), 45 IU; vitamin K_3_ (bisulfate menadione complex), 3 mg; thiamine (thiamine mononitrate), 3 mg; riboflavin, 9 mg; nicotinic acid, 30 mg; pantothenic acid (D-calcium pantothenate), 10 mg; vitamin B_6_, 4 mg; D-biotin, 0.1 mg; folic acid, 2 mg; vitamin B_12_ (cyanocobalamin), 0.02 mg and choline (choline chloride), 1,000 mg.

2 Mineral premix provided the following per kilogram of diet: iron (FeSO_4_•7H_2_O), 55 mg; iodine (Ca (IO_3_)_2_), 1.3 mg; manganese (MnSO_4_•H_2_O), 100 mg; zinc (ZnO), 85 mg; copper (CuSO_4_•5H_2_O), 13 mg; selenium (Na_2_SeO_3_), 0.2 mg.

3 The analyzed values represent the mean of duplicate samples per analysis.

### 2.3 Productive performance

Mortality was weighted and recorded daily, and body weight gain (BWG), average daily feed intake (ADFI), and feed conversion ratio (FCR) were recorded weekly, and the European production efficiency factor (EPEF) was calculated at the end of the experiment (42-d) (17).

### 2.4 Blood collection

On days 25 and 42 of the experiment to determine parameters in serum respectively, 8 birds were selected from each dietary treatment (2 birds per/pen; 1 male and 1 female), and blood samples were obtained from the wing vein after a 2-h feeding withdrawal. The samples were immediately transferred into non-heparinized vacuum tubes, placed at room temperature for 2 h for serum separation and at 1,500 × g/15 min at 4°C for 10 min were centrifuged, and serum without supernatant was removed into vials and was stored at −20°C for and immediately delivered to the laboratory for lipids and glucose analyses. Serum was used to analyze triglyceride (TG, catalog no. 11875540 216, Roche Diagnostic, IN), low-density lipoprotein cholesterol (LDL, catalog no. 03038807 122, plus 2nd generation Kit, Roche Diagnostic, IN), high-density lipoprotein cholesterol (HDL, catalog no. 04713311 190, plus 3rd generation Kit, Roche Diagnostic, Indianapolis, IN), glucose (BG, catalog no. 11876899 216, Glucose Hexokinase Kit, Roche Diagnostic, IN). TG, glucose, total cholesterol, HDL-C, and LDL-C were analyzed using commercial calorimetric diagnostic kits according to the manufacturer’s instructions (Diagnostic Products Corporation, Pars Azmoon, Tehran, Iran). All representative samples were performed twice and measured immediately to avoid variations.

### 2.5 Digestibility assay and calculations

At the end of the grower period (24 d of age), 30 chickens were raised in a metabolic cage (1 bird/cage) for a 4-day preliminary and a 3-day excreta collection (18). In order to determine apparent ileal digestibility (AID), an amount of chromium oxide (3 g/kg of diet) as an indigestible marker was added to each experimental treatment (Celite Hispánica S.A., Alicante, Spain). At the end of the bioassay, five broiler chickens per experimental group were selected and euthanized by cervical dislocation. Ileum segment (Meckel’s diverticulum to 1 cm proximal to the ileocecal junction) was chosen for each bird after the removing of the digestive tract. Samples of fresh digesta from the end half of this section were collected and dried at 55°C for 72 h, and contaminants of scales and feathers were carefully removed before weighing and then ground to pass through a 1.0-mm Wiley mill sieve for subsequent assays. Then, ileal digesta and diet samples were analyzed for DM, CP, EE, and GE as the methods defined by AOAC (19). The DM of diets and ileum samples were defined using a standard procedure of drying the materials in an oven at 105°C/ 24 h (Methods 930.15) and EE content was calculated using the Soxhlet extraction procedure (Method 991.36). The total nitrogen (N) content of diets and ileum samples was measured using the Kjeldahl method. Chromium oxide was determined using Fenton and Fenton (20) procedure. Apparent ileal digestible energy (AIDE) was calculated by multiplying the diet GE content by the apparent ileal energy digestibility (21). The apparent digestibility values of DM, CP, EE, and GE were calculated as follows (22):


$$\mathrm{Digestibility}\;\left(\%\right)=\left(1–\left[\left(Md\times Ni\right)/\left(Mi\times Nd\right)\right]\right)\times 100.$$

where Ni and Mi represent the concentrations of chromium oxide (g/kg DM) in the diet and ileal digesta, respectively; Nd and Md represent the concentration of DM, CP, EE, and GE in the diet and ileal digesta (mg/kg DM), respectively.

### 2.6 Statistical analyses

Prior to conducting the analysis of variance, normality of residues was tested using the Shapiro–Wilk test, while homogeneity of variances was tested using the Levene test. Prior to conducting the analysis of variance, the NORMAL option of the UNIVARIATE procedure and the HOVTEST option were used to determine the normality of residues and variance homogeneity of data, respectively. Data were analyzed in a 2 × 2 × 2 factorial arrangement of treatments based on completely randomized design were subjected to One-Way Analysis of Variance (ANOVA) using the general linear models (GLM) procedure of SAS® software (SAS Institute, 2009, version 9.4) to determine the main effects of the source of oils (soy oil and palm oil), oil levels (3 and 6%), and LP (0 and 0.1%), and all two-way interactions. If significant main effects or interactions were observed, Tukey’s test was used to compare differences among the treatment means. The significance level was set at *p* < 0.05 for all statistical analyses.

## 3 Results

### 3.1 Growth performance

Mortality was low (<1.5%), and no appreciable differences in mortality were observed among treatments. The effects of oil sources and levels, as well as LP supplementation on growth performance during 1 to 21 and 22 to 42 d of age, and the whole period are shown in Tables 2, 3, respectively. Broilers fed the SO-containing diets had statistically higher BWG, ADFI, and EPEF in all the periods (1 to 21, 22 to 42, and 1 to 42 d) compared to those fed the PO-containing diets (*p <* 0.01). Supplementation of LP into the diets improved BWG, FCR, and EPEF in broilers from 22 to 42 d and the entire period of the experiment (*p <* 0.01). From 22 to 42 and 1 to 42 d, broilers fed the SO-containing diets had better FCR compared with those fed the PO-containing diets (*p <* 0.05). There was a significant interaction between LP supplementation and oil levels on ADFI from 22 to 42 d, and FCR from 22 to 42 d and the whole period (*p <* 0.05). The LP supplementation to diets containing 6% oil decreased ADFI and improved FCR in broilers (*p <* 0.05).

**Table 2:** Effect of different oil sources, oil inclusion levels, and lysophospholipid supplementation on the performance of broiler chickens during 1 to 21 and 22 to 42 d of age.

Item	1–21 d			22–42 d		
	BWG (g/d)	ADFI (g/d)	FCR	BWG (g/d)	ADFI (g/d)	FCR
Oil sources						
Soybean oil	38.4^a^	56.3^a^	1.47	84.9^a^	161.2^a^	1.90^b^
Palm oil	36.3^b^	54.1^b^	1.49	79.2^b^	156.2^b^	1.97^a^
Oil levels						
3%	37.2	55.0	1.48	81.7	158.1	1.94
6%	37.4	55.4	1.48	82.4	159.3	1.94
LP						
0 mg/kg	37.1	54.7	1.48	80.7^b^	160.1	1.99^a^
100 mg/kg	37.5	55.7	1.49	83.4^a^	157.3	1.89^b^
SEM^1^ (*n* = 16)	0.47	0.50	0.014	0.59	1.06	0.019
Oil level × LP						
3 × 0	37.6	54.9	1.46	80.3	156.8^b^	1.96^ab^
3 × 100	36.8	55.2	1.51	83.2	159.5^ab^	1.92^ab^
6 × 0	36.6	54.6	1.49	81.2	163.5^a^	2.02^a^
6 × 100	38.3	56.2	1.47	83.5	155.1^b^	1.86^b^
SEM (*n* = 8)	0.66	0.71	0.019	0.85	1.50	0.027
*p*-value						
Oil source	0.004	0.003	0.274	0.001	0.003	0.012
Oil level	0.720	0.622	0.925	0.479	0.443	0.971
LP	0.547	0.193	0.520	0.005	0.067	0.001
Oil source × Oil level	0.078	0.215	0.176	0.461	0.995	0.593
Oil source × LP	0.569	0.885	0.322	0.532	0.405	0.720
Oil level × LP	0.072	0.346	0.098	0.766	0.001	0.033
Oil source × Oil level × LP	0.158	0.364	0.234	0.601	0.466	0.973

^a, b^Means in the same column followed by different letters are significantly different (*p <* 0.05).

1 SEM, pooled standard error of the mean.

**Table 3:** Effect of different oil sources, oil inclusion levels, and lysophospholipid supplementation on the performance of broiler chickens from 1 to 42 days of age.

Item	BWG (g/d)	ADFI (g/d)	FCR	42 d-BW (g)	Mortality (%)	EPEF^2^
Oil sources						
Soybean oil	61.6^a^	108.8^a^	1.77^b^	2632^a^	0.94	295.3^a^
Palm oil	57.8^b^	105.1^b^	1.82^a^	2469^b^	1.06	264.4^b^
Oil levels (%)						
3	59.5	106.6	1.79	2,542	0.93	275.7
6	59.9	107.3	1.79	2,560	1.06	284.0
LP						
0 mg/kg	58.9^b^	107.4	1.82^a^	2519^b^	1.06	266.0^b^
100 mg/kg	60.4^a^	106.5	1.76^b^	2583^a^	0.94	293.7^a^
SEM^1^ (*n* = 16)	0.41	0.62	0.015	17.2	0.207	6.25
Oil level × LP						
3 × 0	59.0	105.8	1.80^ab^	2,520	1.00	266.1
3 × 100	60.1	107.3	1.79^ab^	2,563	0.88	285.2
6 × 0	58.9	108.0	1.85^a^	2,417	1.13	265.9
6 × 100	60.8	105.6	1.74^b^	2,602	1.00	302.2
SEM (*n* = 8)	0.58	0.88	0.021	24.3	0.293	8.84
*p*-value						
Oil source	0.001	0.001	0.013	0.001	0.674	0.001
Oil level	0.469	0.394	0.974	0.473	0.674	0.354
LP	0.015	0.283	0.007	0.015	0.674	0.005
Oil source × Oil level	0.607	0.616	0.940	0.621	0.402	0.625
Oil source × LP	0.435	0.512	0.982	0.433	0.402	0.550
Oil level × LP	0.402	0.110	0.013	0.399	0.987	0.339
Oil source × Oil level × LP	0.234	0.324	0.689	0.236	0.674	0.872

^a, b^Means in the same column followed by different letters are significantly different (*p <* 0.05).

1 SEM, pooled standard error of the mean.

2 EPEF = BW (kg) × % liveability × 100/FCR × trial duration (d).

### 3.2 Ileal digestibility of nutrients

Nutrient digestibility data showed that the inclusion of soybean oil and palm oil in the broiler diet had a different effect on apparent digestibility of DM and EE, and AIDE of diets from 18 to 21 d of age (Table 4). The AID of DM and EE, and AIDE values for diets containing SO were greater than those of diets containing PO (*p <* 0.05). The EE digestibility and AIDE were greater in the diets containing 6% oil compared with the diets with 3% oil (*p <* 0.05). No significant differences between diets were observed for the AID coefficients of CP (*p >* 0.05). The LP supplementation to the diet improved the apparent digestibility of DM and EE, and AIDE, whereas a significant interaction between LP × Oil levels was observed on EE digestibility and AIDE (*p <* 0.05). Supplementation of LP to the diets containing 6% oil increased apparent digestibility EE, and AIDE in broilers from 18 to 21 d of age (*p <* 0.05).

**Table 4:** Effect of different oil sources, oil inclusion levels, and lysophospholipid supplementation on apparent ileal digestibility (AID) of nutrients (%).

Item	DM (%)	CP^2^ (%)	EE (%)	AME (kcal/kg)
Oil sources				
Soybean oil	71.35^a^	80.37	81.97^a^	3204^a^
Palm oil	65.82^b^	80.19	77.62^b^	3153^b^
Oil levels (%)				
3	68.49	80.48	78.37^b^	3168^b^
6	68.67	80.07	81.22^a^	3189^a^
LP				
0 mg/kg	67.92^b^	80.67	78.63^b^	3169^b^
100 mg/kg	69.24^a^	79.89	80.96^a^	3188^a^
SEM (*n* = 16)	0.336	0.772	0.549	2.0
Oil level × LP				
3 × 0	67.84	81.43	78.18^b^	3166^b^
3 × 100	69.14	79.55	78.56^b^	3171^ab^
6 × 0	68.00	79.92	79.09^ab^	3173^ab^
6 × 100	69.35	80.23	83.36^a^	3205^a^
SEM^1^ (*n* = 8)	0.476	1.091	1.143	2.8
*p*-value				
Oil source	0.001	0.877	0.001	0.001
Oil level	0.701	0.708	0.001	0.001
LP	0.010	0.477	0.006	0.001
Oil source × Oil level	0.547	0.698	0.337	0.469
Oil source × LP	0.652	0.397	0.206	0.548
Oil level × LP	0.964	0.326	0.020	0.001
Oil source × Oil level × LP	0.971	0.888	0.113	0.425

^a, b^Means in the same column followed by different letters are significantly different (*p <* 0.05).

1 SEM, pooled standard error of the mean.

2 CP = N × 6.25.

### 3.3 Serum metabolite parameters

On day 25, blood serum TG and LDL-C contents were lower, while HDL-C was higher in both male and female broilers fed SO-containing diets than those fed PO-containing diets (Table 5; *p <* 0.01). However, LP supplementation to broiler diets showed no significant effect on serum lipid variables (*p >* 0.05). At 25 days, birds fed diets containing 6% oil had greater TG than birds fed diets containing 3% oil (*p <* 0.05).

**Table 5:** Effect of different oil sources, oil inclusion levels, and lysophospholipid supplementation on serum lipids and glucose concentration at 25 days of age.

Item	TG (mg/dl)		Total cholesterol (mg/dl)		HDL-C (mg/dl)		LDL-C (mg/dl)		Glucose (mg/dl)	
	Male	Female	Male	Female	Male	Female	Male	Female	Male	Female
Oil sources										
Soybean oil	41.8^b^	41.7^b^	104.1	107.5	76.2^a^	75.9^a^	16.5^b^	23.2^b^	213.1	219.6
Palm oil	56.6^a^	59.1^a^	103.5	106.7	68.8^b^	66.6^b^	23.4^a^	30.3^a^	210.9	218.2
Oil levels (%)										
3	44.4^b^	45.6^b^	103.6	107.2	73.0	69.3	21.7	28.7	212.6	219.0
6	53.9^a^	54.3^a^	103.9	107.1	75.1	71.3	18.1	24.7	211.5	218.9
LP										
0 mg/kg	49.9	51.1	104.1	107.6	74.7	71.0	19.3	26.4	211.3	217.5
100 mg/kg	48.5	49.8	103.5	106.7	73.3	69.6	20.5	27.1	212.8	220.4
SEM^1^ (*n* = 16)	0.65	0.55	1.74	1.20	0.97	0.71	2.08	1.44	3.61	2.69
*p*-value										
Oil source	0.001	0.001	0.829	0.631	0.001	0.001	0.027	0.002	0.678	0.707
Oil level	0.001	0.001	0.899	0.935	0.151	0.068	0.227	0.059	0.825	0.972
LP	0.141	0.111	0.837	0.618	0.298	0.199	0.675	0.717	0.761	0.447
Oil source × Oil level	0.082	0.086	0.724	0.478	0.722	0.503	0.740	0.425	0.556	0.278
Oil source × LP	0.787	0.953	0.843	0.624	0.688	0.262	0.969	0.884	0.217	0.174
Oil level × LP	0.745	0.525	0.791	0.518	0.746	0.817	0.679	0.633	0.624	0.622
Oil source × Oil level × LP	0.822	0.902	0.754	0.744	0.416	0.348	0.533	0.444	0.840	0.793

^a, b^Means in the same column followed by different letters are significantly different (*p <* 0.05).

1 SEM, pooled standard error of the mean.

On day 42, both male and female broilers fed SO-containing diets had higher serum HDL-C concentrations and lower TG and LDL-C concentrations than those fed PO-containing diets (Table 6; *p <* 0.01). The LP supplementation to broiler diets reduced serum TG, total cholesterol, and LDL-C concentrations, whereas enhanced HDL-C concentrations at 42 days of age compared to unsupplemented diets (*p <* 0.05). On day 42, male and female broilers fed diets containing 6% oil had higher TG than those fed diets containing 3% oil (*p <* 0.05).

**Table 6:** Effect of different oil sources, oil inclusion levels, and lysophospholipid supplementation on serum lipids and glucose concentration at 42 days of age.

Item	TG (mg/dl)		Total cholesterol (mg/dl)		HDL-C (mg/dl)		LDL-C (mg/dl)		Glucose (mg/dl)	
	Male	Female	Male	Female	Male	Female	Male	Female	Male	Female
Oil sources										
Soybean oil	52.2^b^	47.0^b^	112.8	118.4	86.2^a^	90.6^a^	16.1^b^	18.5^b^	229.7	238.2
Palm oil	62.4^a^	63.0^a^	114.5	120.6	72.7^b^	76.0^b^	29.3^a^	32.0^a^	229.5	237.7
Oil levels (%)										
3	52.3^b^	50.3^b^	111.8	117.4	78.9	82.4	22.3	25.0	231.5	240.1
6	62.4^a^	59.7^a^	115.5	121.7	80.0	84.3	23.0	25.5	227.7	235.7
LP										
0 mg/kg	61.4^a^	58.7^a^	117.5^a^	123.5^a^	76.5^b^	79.8^b^	28.7^a^	31.9^a^	228.1	236.3
100 mg/kg	53.2^b^	51.3^b^	109.8^b^	115.6^b^	82.4^a^	86.8^a^	16.6^b^	28.6^b^	231.0	239.5
SEM^1^ (*n* = 16)	0.95	0.78	2.59	1.84	0.83	0.72	2.86	2.11	3.52	2.72
*p*-value										
Oil source	0.001	0.001	0.655	0.420	0.001	0.001	0.003	0.001	0.960	0.901
Oil level	0.001	0.001	0.317	0.115	0.380	0.075	0.867	0.876	0.458	0.264
LP	0.001	0.001	0.045	0.006	0.001	0.001	0.006	0.001	0.556	0.402
Oil source × Oil level	0.395	0.384	0.752	0.711	0.087	0.091	0.608	0.376	0.860	0.624
Oil source × LP	0.786	0.753	0.942	0.947	0.188	0.063	0.628	0.450	0.547	0.563
Oil level × LP	0.928	0.912	0.925	0.980	0.542	0.925	0.927	0.989	0.871	0.947
Oil source × Oil level × LP	0.690	0.563	0.717	0.515	0.462	0.777	0.884	0.619	0.518	0.287

^a, b^Means in the same column followed by different letters are significantly different (*p <* 0.05).

1 SEM, pooled standard error of the mean.

## 4 Discussion

Oils are needed for the proper functioning of the body, as they protect against stress, help maintain body temperature, facilitate hormone synthesis, support suitable central nervous system function, and enhance muscle metabolism performance (23). The Fat-enhanced feeds elevate the efficiency of the consumed energy and productivity in poultry under normal and hot climate condition, but the impact depends on fat/oil source (24). The study revealed that broilers fed diets containing soybean oil exhibited better BWG, FI, and FCR than those that were fed diets containing palm oil. It is suggested that broiler chickens perform better when fed with plant oil sources such as soybean (25). These observations are consistent with the findings of Dänicke et al. (26). Furthermore, adding an emulsifier to diets containing 6% oil resulted in decreased DFI and improved feed conversion ratio. The study found a significant interaction between LP supplementation and oil levels in relation to FI and FCR. However, the LP emulsifier supplement in a basal diet containing 6% oil has demonstrated relatively higher performance compared to a 3% oil source in growing broiler chickens in the grower and throughout the entire production period. Since oils create a coating around nutrients, hindering the optimal function of digestive enzymes, emulsifiers indirectly improve the digestibility of fats and subsequently enhance the digestibility of other nutrients and organic matter (27). It has been reported that broilers fed 6% soybean oil (mostly 84% an unsaturated oil) or a combination of soybean oil and palm oil (mostly 50% a saturated oil) exhibit significantly increased growth during days 1–21 of age compared to a control diet with 6% palm oil (24).

Birds can control their feed intake according to their needs, the type of diet, and environmental conditions. The primary factor that determines satiety in birds is the amount of energy they obtain from their diet (28, 29). Feed intake continues until a physical or physiological satiety factor prevents further feed consumption by the bird. A reduction in feed intake may lead to a decrease in feed conversion ratio, which could be attributed to the availability of adequate energy required by the diets due to the inclusion of emulsifiers. This decrease in feed intake is probably because the high energy requirements needed to support rapid growth rates are usually met in low-energy diets (30). It has been found that after 21 days, the use of emulsifiers can increase the concentration of bile salts in the intestine, which can act as a competitive inhibitor and reduce the activity of lipase enzymes and colipase on fat digestion. This can lead to increased fat retention time in the digestive tract, decreased feed passage rate, and ultimately decreased feed consumption (31). Moreover, under heat stress conditions, growth, respiratory system and cardiovascular was found to be adversely affected by increasing dietary protein concentration, which causes low performance (1). High environmental temperatures stimulate the peripheral thermal receptors to transmit suppressive nerve impulses to the appetite center in the hypothalamus. The result is a reduction in the consumption of feed and thus of nutrients available for heat dissipation, hormone synthesis and enzymatic activities (3).

During heat stress, feed intake decreases. Increased stimulation of heat receptors at high environmental temperatures leads to the suppression of neural impulses to the appetite center in the hypothalamus, resulting in reduced appetite. Decreased feed intake during heat stress is one of the reasons for reduced performance in broiler chickens (32). Additionally, heat stress has an impact on the digestion and absorption of feed as a result of decreased blood flow in the gastrointestinal system. This can potentially lead to damage to the intestinal epithelial tissue and increase the likelihood of bacterial pathogens and endotoxins entering the bloodstream, which can subsequently cause severe inflammation and reduce the digestion and absorption of nutrients (33, 34). In this study, during a period of poultry rearing, they experienced heat stress. However, the birds that received an emulsifier in their diet performed better. This improvement can be attributed to enhanced energy availability and fatty acids, which positively influenced performance despite reduced feed intake.

Losses in productivity of broilers caused by continuous heat exposure could be explained by the decrease in nutrients digestibility that may be caused by peripheral vasodilation and reduced blood flow in the gut and thus decreased intestinal function (1). Lysophosphatidylcholine emulsifiers have been shown to significantly increase intestinal villus height in birds (35, 36). This demonstrates that this type of emulsifier supplement can improve nutrient absorption, enhance nutrient metabolism, and have a positive impact on growth. Adding emulsifiers to the diet of broiler chickens in the early stages has greater importance because the activity of the lipase enzyme in chicks usually peaks between 40 and 56 days of age (37). Furthermore, Zhang et al. (27) also reported that phosphatidylcholine emulsifiers can increase the lipid cycle in poultry, reduce abdominal fat, improve fat deposition in muscles and muscle quality, increase body weight, and improve feed conversion ratio in broiler chickens (27, 38, 39). On the other hand, the reason for the increase in body weight with phosphatidylcholine emulsifier supplementation in a diet containing 6% palm oil throughout the rearing period may be due to the synchronization of this emulsifier with the lipolytic enzymes that break down fat from the first day of birth until the end of rearing periods. As lysophosphatidylcholine has a higher hydrophilic property compared to regular phospholipids and bile acids, it can enhance fat digestion and absorption in young birds, thereby positively impacting growth performance during production (40). Lysophosphatidylcholine can spontaneously form very small micelles in the intestine because it reaches a critical micelle concentration of 0.02–0.20 millimolar per liter, which is 20–200 times more effective than bile salts secreted by the liver. This indicates the superior effects of lysophosphatidylcholine compared to bile salts and lecithin in terms of emulsification capacity and micelle formation, which also affects other nutrients present in digestion. Therefore, it can be considered an effective exogenous emulsifier and a better source of emulsification to enhance the performance of birds (12, 41). The improvement in growth performance in the current study may also be related to the enhanced utilization of crude protein and fat. San Tan et al. (42) and Zhang et al. (12) also reported that incorporating emulsifiers in the diet of broiler chickens improves fat digestion and absorption, as well as nutrient digestibility, resulting in increased performance. Emulsifiers increase the incorporation of micelles in the intestinal lumen, thereby enhancing fat digestion (43). In this study, the apparent digestibility coefficients of ether extract, dry matter, and apparent ileal digestible energy were affected by the source and level of oil and emulsifier in the diet. The apparent ileal digestibility of ether extract was higher in birds fed diets containing soybean oil. It has been reported that bile salts, by emulsifying fats, create smaller-sized particles that lead to decreased activity of the enzyme lipase. Therefore, it appears that in the current study, emulsifiers have generated smaller particles of fats, resulting in enhanced secretion of lipase enzymes (44). The limited digestibility of fats in young chicks is due to their low bile production (42). It is likely that when oil is added to the diet, the amount of bile salts produced by the chicks is insufficient, or some of the bile salts are degraded and become unavailable for the bird’s use by the digestive microorganisms (45). Emulsifiers have been recognized for their ability to improve fat digestion and support birds in overcoming the inefficiency of lipase before 40 days of age in broiler chicks (46). In contrast to our findings, Zhang et al. (12) and Kavouridou et al. (47) reported that the digestibility of nutrients was not significantly affected in broiler chicks fed diets containing palm oil, soybean oil, and flaxseed oil. The intestinal digestibility of ether extract in the broilers fed diets containing soybean oil was higher. This increased digestibility of the ethereal extract may be attributed to the fatty acids profile and the presence of free fatty acids in soybean oil. Palm oil has a high concentration of palmitic and stearic acids. As a percentage of total saturated fatty acids, palm oil contains palmitic acid (88%) and stearic acid (9%). Stearic acid is an inhibitor of lipase activity and high concentrations of stearic acid in palm oil may inhibit lipase activity, resulting in lower apparent metabolizable energy (AME) and digestibility of nutrients (48). Previous findings have demonstrated that free fatty acids can bind to the mineral calcium and reduce its bioavailability for meat-producing and egg-laying chickens (49, 50). It can be inferred that the supplementation of phosphatidylcholine emulsifier enhances the availability of smaller fat droplets, thereby improving the performance of lipase and subsequently enhancing the digestibility of the ether extract in broiler chicks receiving high levels of dietary fat.

Blood profiles can be used as a marker of health status and malnutrition in poultry. The study revealed that incorporating soybean oil into broiler diets improved the lipid profile, with lower levels of TG and LDL-C and higher levels of HDL-C. Additionally, broilers fed diets containing a higher level of oil exhibited elevated TG levels. Nowadays, the existence of a relationship between the concentration of blood serum lipids (cholesterol, triglycerides, HDL-C, VLDL, and LDL-C) and cardiovascular diseases in humans has been proven. The metabolic profiles showed negative effects from heat stress; e.g., total cholesterol and glucose. The adverse influence of the Heat stress on the productive traits of broilers was associated with increased blood glucose and stress index (H/L ratio) ratio and a decrease in the internal innate immunity. In addition, a decrease in the digestibility of crude protein and crude fiber were found. This could be due to the increased heat increment of protein, the reduction in energy availability for maintenance and heat dissipation, and a decrease in nutrient digestibility that may be caused by peripheral vasodilation and reduced blood flow in the digestive system (1). Increased LDL concentration in the blood due to the use of plant oils such as palm oil leads to the development of arterial blockage and hardening of the artery walls. Furthermore, elevated levels of serum triglycerides increase the likelihood of cardiovascular diseases. Although the impact of dietary lipids on the occurrence of cardiovascular diseases in poultry is not of significant importance due to their short economic lifespan, it is crucial due to the effects of blood lipids on the quality of the end product that eventually reaches human consumption (51). Similarly, Siyal et al. (10) demonstrated that the LDL-C concentration decreased in the soy lecithin group (1.0%) compared to the control diet in broilers, while the HDL-C concentration improved, possibly due to better emulsification of emulsifiers, which can effectively and completely impact oils. HDL has an important antioxidant effect that not only inhibits phospholipid oxidation but also reduces LDL activity (52).

In this study, the addition of LP (Lecithin Phospholipid Complex) to diets resulted in a significant reduction in serum TG, total cholesterol, and LDL-C concentrations, while simultaneously increasing HDL-C concentrations at 42-d. Cho et al. (53) utilized a 0.5% emulsifier in a low-energy diet and reported a decrease in blood triglyceride levels. They stated that emulsifiers break down triglycerides for energy utilization. Based on this, it can be concluded that emulsifiers effectively reduce blood triglyceride concentrations by utilizing energy with increasing bird age. Emulsifiers can also lower the overall plasma cholesterol levels by reducing the secretion of lipoprotein molecules in the blood (54).

In addition, glucose depend upon insulin and its signaling effects to get into cells. Therefore, it seems that an increased blood level of LP competes with TG to enter cells, and as a result, it decreases the concentration of TG in the blood. Dietary LP supplementation had no significant effect on serum glucose concentration (55). Boontiam et al. (56) found the increasing blood glucose concentration accompanied by increased supplementation level of LP. They believed that dietary LP supplementation stimulated uptake of glucose, which is commonly used as an energy source and a metabolic intermediate. The increased availability of glucose was also detected in birds fed with glycerol polyethylene glycol-ricinoleate as a synthetic emulsifier (56). Based on the report by Wangkahart et al. (57), there was no significant effect of dietary supplemental emulsifier on serum concentration of total protein. Lysophospholipids (LP) can alter protein channel formation in the membrane by increasing ion exchanges (58). This change increases the number and size of the membranous pores and improves the flux rate of macromolecules across the cell membrane consequently. Both mechanisms can induce nutrient transport (55).

The results showed that birds fed LP supplementation increased concentrations of HDL and body weight gain in broilers. It is commonly established that the HDL plays an important function in carrying total cholesterol and free fatty acids from the body’s tissue to the hepatocytes for excretion or re-metabolization. The increased level of HDL with LPL supplementation indicated the clearance rate of body cholesterol, which possibly affected hepatic lipogenesis of broilers (59). The improvement in weight gain level is usually indicated as an available uptake of glucose, which enhances the metabolic pathway in the hepatocytes (60).

The potential effect of LP supplementation addition on body weight gain concentration detected in this study would alter the metabolism of glucose in order to supply nutrients for their production needs. These findings highlight the potential of emulsifier supplements to enhance nutrient absorption, improve nutrient metabolism, and promote growth even under heat-stress conditions.

## 5 Conclusion

The study found that broiler chickens fed soybean oil had better weight gain, feed intake, and feed conversion ratio than those fed palm oil. Emulsifier enhanced fat digestion, nutrient absorption, and growth performance, particularly in the early stages of rearing. Supplementation of 100 mg/kg lysophospholipid emulsifiers helped mitigate the negative effects of heat stress on feed intake and nutrient absorption, resulting in improved performance. Overall, Supplementation of 100 mg/kg lysophospholipid emulsifiers to the diets containing 6% oil, particularly LP, in broiler diets can boost growth, nutrient digestion, and lipid metabolism, even under challenging conditions like heat stress.

## Data Availability

The raw data supporting the conclusions of this article will be made available by the authors, without undue reservation.

## Glossary

T3: tri-iodothyronine
T4: tetra-iodothyronine
HLB: hydrophilic–lipophilic balance
LP: Lysophospholipids
HS: Heat Stress
BWG: body weight gain
ADFI: average daily feed intake
FCR: feed conversion ratio
EPEF: European production efficiency factor
FI: feed intake
TG: triglyceride
LDL: low-density lipoprotein
HDL: high-density lipoprotein cholesterol
AID: apparent ileal digestibility
AME: apparent metabolisable energy
CP: crude protein
DM: dry matter
EE: ether extract
N: nitrogen
AIDE: Apparent ileal digestible energy
GE: gross energy
ANOVA: one-way analysis of variance
GLM: general linear models
H/L: heterophils to lymphocyte
LP: Lecithin Phospholipid
HLB: hydrophilic–lipophilic balance
SO: soybean oil
PO: palm oil
